# Correction: The antimicrobial activity of an antiseptic soap against *Candida Albicans* and *Streptococcus Mutans* single and dual-species biofilms on denture base and reline acrylic resins

**DOI:** 10.1371/journal.pone.0320387

**Published:** 2025-03-13

**Authors:** Camilla Olga Tasso, Beatriz Ribeiro Ribas, Túlio Morandin Ferrisse, Paula Aboud Barbugli, Jonatas Silva de Oliveira, Janaina Habib Jorge

The scientific names “*Candida Albicans*” and “*Streptococcus Mutans*” are formatted incorrectly in the article title. The correct title is: The antimicrobial activity of an antiseptic soap against *Candida albicans* and *Streptococcus mutans* single and dual-species biofilms on denture base and reline acrylic resins.

Paula Aboud Barbugli is not included in the author byline. Paula Aboud Barbugli should be listed as the fourth author and affiliated with the Department of Dental Materials and Prosthodontics, São Paulo State University (Unesp), School of Dentistry, Araraquara, São Paulo, Brazil. The contribution of this author is: Development of methodology.

The author initials appear incorrectly in the citation.

The correct citation is: Tasso CO, Ribas BR, Ferrisse TM, Barbugli PA, de Oliveira JS, Jorge JH (2024) The antimicrobial activity of an antiseptic soap against *Candida albicans* and *Streptococcus mutans* single and dual-species biofilms on denture base and reline acrylic resins. PLoS ONE 19(7): e0306862. https://doi.org/10.1371/journal.pone.0306862.

In the Abstract section, there is an error in the first sentence. The correct sentence is: The objective of this study was to evaluate the effect of antiseptic soap on single and dual-species biofilms of Candida albicans and Streptococcus mutans on denture base and reline resins.

In the Assessment of cell viability by CLSM subsection of Materials and methods, there is an error in the second sentence. The correct sentence is: Afterward, 10 mL of saline solution containing 2 mL of live/dead dye (SYTO-9 and propidium iodide (PI) from Molecular Probes (Eugene, OR, USA) was added to the samples.

There is an error in the caption for Figs 4 and 6. Please see the complete, correct Figs 4 and 6 caption here.

**Fig 4:**
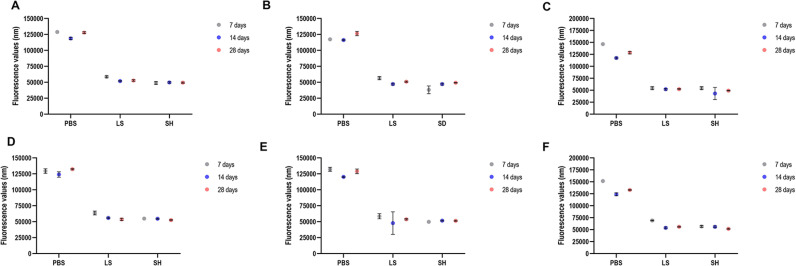
Results of the cellular metabolism of the C. albicans and S. mutans cells (single and dual-species biofilms) referring to biofilm formation on denture base (A-C) and reline acrylic resin (D-F) samples after immersion in PBS, Lifebuoy, and 0.5% sodium hypochlorite solutions. A and D: C. albicans; B and E: S. mutans; C and F: dual-species biofilm.

**Fig 6:**
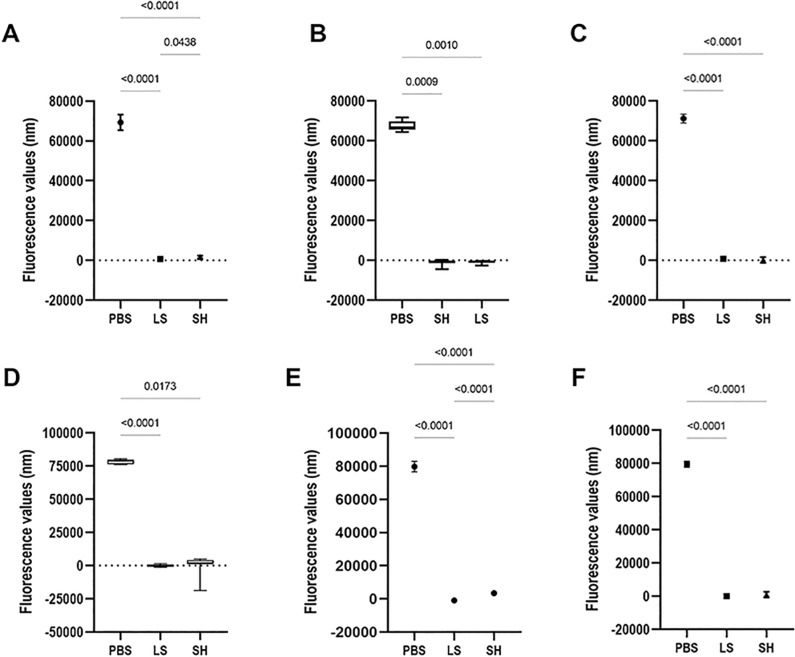
Results of the cellular metabolism of the C. albicans and S. mutans cells (single and dual-species biofilms) formed on denture base (A-C) and reline acrylic resins (D-F) samples. A and D: C. albicans; B and E: S. mutans; C and F: dual-species biofilm.

In the Prevention protocol subsection of the Results, there is an error in fourth sentence of the seventh paragraph. The correct sentence is: A significant reduction in cell metabolism was observed in the S. mutans single biofilm for both the Lifebuoy and sodium hypochlorite groups compared to the PBS group (Fig 4E).
