# Supplementary material for: The antimicrobial activity of an antiseptic soap against Candida Albicans and Streptococcus Mutans single and dual-species biofilms on denture base and reline acrylic resins
Source: PLoS One. 2024 Jul 11;19(7):e0306862. doi: 10.1371/journal.pone.0306862 (PMC11239035; doi:10.1371/journal.pone.0306862)
Supplement: S1 File — (DOCX) [file pone.0306862.s001.docx]

**RESULTS**

**Prevention protocol (individual measures)**

**Microbial viability (UFC/mL)**

Denture base acrylic

Hard relining

Denture base acrylic

Hard relining

**Cellular metabolism (Alamar Blue assay)**

Denture base acrylic

Hard relining

**Desinfection protocol (individual measures)**

**Microbial viability (UFC/mL)**

**Cellular metabolism (Alamar Blue assay)**

Denture base acrylic

Hard relining
